# Impact of bariatric surgery on maternal gestational weight gain and pregnancy outcomes in women with obesity: A population-based cohort study from Qatar

**DOI:** 10.5339/qmj.2024.2

**Published:** 2024-01-22

**Authors:** Jesni Vazhiyelethil, Fathima Minisha, Sawsan Al Obaidly, Mai AlQubaisi, Husam Salama, Najah Ali, Najat Khenyab, Suruchi Mohan, Abdul Rouf Pallivalappil, Nader Al-Dewik, Hilal Al Rifai, Tom Farrell

**Affiliations:** Department of Obstetrics and Gynecology, Women's Wellness and Research Centre, Hamad Medical Corporation, Doha Qatar. Email: fminisha@hamad.qa; Department of Pediatrics and Neonatology, Women's Wellness and Research Centre, Hamad Medical Corporation, Doha, Qatar; Department of Obstetrics and Gynecology, Sidra Medicine, Doha Qatar; Department of Research, Women's Wellness and Research Centre, Hamad Medical Corporation, Doha Qatar; Chief Executive Officer, Women's Wellness and Research Centre, Hamad Medical Corporation, Doha, Qatar

**Keywords:** Bariatric surgery, gestational weight gain, cesarean delivery

## Abstract

**Background:**

Bariatric surgery is performed in obese women of reproductive age to help achieve a healthy prepregnancy weight to reduce the complications associated with obesity in pregnancy. However, these procedures can impact maternal nutrition and gestational weight gain (GWG). This study evaluates the maternal and neonatal outcomes in women with prepregnancy bariatric surgery and determines the impact on GWG.

**Methods:**

This study included 24 weeks gestation or more pregnancies, with a maternal BMI at delivery of 30 kg/m^2^ or more. It was categorized into two groups based on whether they had prepregnancy bariatric surgery (exposed) or not (unexposed). The outcomes included gestational diabetes (GDM), gestational hypertension (GHT), mode of delivery, preterm birth (PTB), GWG, birthweight (BW) and customized BW centiles, low birthweight (LBW), congenital anomalies, and admission to the neonatal intensive unit (NICU). Categorization was also done based on the adequacy of GWG (low, adequate, and excess).

**Results:**

A total of 8,323 women were included in the study, 194 of whom had prepregnancy bariatric surgery. After adjusting for confounders, the exposed group had a mean GWG 1.33 kg higher than the unexposed group (95% CI 0.55-2.13, p = 0.001). The exposed group had higher odds of PTB (aOR 1.78, 95% CI 1.16-2.74, p = 0.008), CD (aOR 6.52, 95% CI 4.28-9.93, p < 0.001), LBW in term babies (aOR 2.60, 95% CI 1.34-5.03, p = 0.005), congenital anomalies (aOR 2.64, 95% CI 1.21-5.77, p = 0.015), low APGAR score (aOR 3.75, 95% CI 1.12-12.5, p = 0.032) and 80.4g lesser birthweight (95% CI -153.0, -5.8; p = 0.034). More women in the low GWG category had LBW babies (28.6% versus 6.7% in the high GWG group, p = 0.033), lowest mean BW and median BW centiles (2775 grams versus 3289 grams in the high GWG group, p = 0.004 and 57.5% versus 74.5% in the high GWG group, p = 0.040, respectively).

**Conclusion:**

The findings of this study highlight differences in perinatal outcomes such as preterm birth, low birth weight, congenital anomalies, cesarean deliveries, and gestational weight gain between post-bariatric women and controls. These insights can help inform the planning and provision of appropriate maternity care to enhance patient safety and outcomes. The results of this study can also guide the counseling of reproductive age-group women who are planning to undergo bariatric surgery.

## Introduction

The global rise in obesity is unrelenting, specifically in women of reproductive age ^1,2^, with around 1 in 3 being overweight or obese in the UK and the US ^3,4,5^. In Qatar, obesity rates (body mass index higher than 30 kg/m^2^) among childbearing women are as high as 38%^6^. The impact of maternal obesity on pregnancy and perinatal outcomes is well-established^7^. There is an increased risk of pregnancy complications, including gestational diabetes, pre-eclampsia, preterm and post-term birth, cesarean delivery, large or small for gestational age infants, congenital anomalies, and increased perinatal mortality^8,9,10^.

Bariatric surgery is an established treatment option for long-term weight loss, with over 50% of all surgeries performed on women of reproductive age^11,12^. They can be classified into two types: malabsorptive or restrictive. Malabsorptive procedures such as Roux-En-Y bypass and biliopancreatic diversion reduce micronutrient absorption due to small bowel bypass. In contrast, restrictive procedures such as laparoscopic adjustable gastric banding and sleeve gastrectomy reduce gastric capacity and have less impact on nutrient absorption^13^.

Current literature, including systematic reviews, generally reports improved maternal and perinatal outcomes following bariatric surgery; however, this evidence is often based on small, often low-quality studies with high levels of heterogeneity between the studies included^14,15^. The determinants of obesity and associated lifestyle choices, however, may persist after bariatric surgery, and in the context of pregnancy, these factors may influence weight gain during pregnancy. Excessive gestational weight gain has been associated with less favorable pregnancy outcomes^16^. However, data on the effect of weight gain in pregnancies post-bariatric surgery are limited^17^ and need further exploration.

The aim of this population-based study, using data from a hospital registry, was to compare the maternal and perinatal outcomes in pregnancies of two groups of women with a BMI equal to or greater than 30 kg/m^2^ at the time of delivery: those who had had a previous bariatric weight loss procedure and a control group who had no prior history of weight loss surgery. Further exploration of gestational weight gain and its association with pregnancy outcomes in the two groups was performed.

## Materials And Methods

### Study design and setting

This study constitutes a secondary data analysis in the 2017-2018 Qatar PEARL-Peristat study registry (Perinatal Neonatal Outcomes Research Study in the Arabian Gulf). This population-based registry, funded by the Qatar National Research Fund (Grant number: NPRP 6-238-3-059), collected comprehensive hospital data concerning women delivering in government hospitals in Qatar and their offspring. The Hamad Medical Corporation (HMC) Institutional Review Board (HMC-IRB 13064/13) approved the registry with a waiver of consent, providing blanket approval for all secondary data analyses conducted from the registry.

### Participants

This study encompassed all live births at or beyond 24 weeks gestation, with mothers having a BMI of 30 kg/m^2^ or more at parturition, and delivered between April 2017 and March 2018 at Qatar's largest tertiary care facility. Women were then categorized into two groups based on whether they had documented evidence of bariatric surgery before pregnancy or not. No other exclusion criteria were applied. While the registry did not report the specific type of bariatric surgery, in Qatar, laparoscopic restrictive sleeve gastrectomy is the most frequently performed procedure in HMC, accounting for nearly 95% of bariatric surgeries^18^.

### Data source and variables

After applying the inclusion criteria, all data for this study were extracted from the PEARL registry, which documented every delivery at the tertiary maternity hospital during the study period. Only the data of women delivering at this largest tertiary center were extracted from the national registry. Independent data collectors collected the data in the registry from patient health records. Only anonymized data was extracted for analysis to maintain confidentiality.

The maternal demographic variables included age at delivery in completed years (dichotomized using a cutoff of 30 years), height, weight at booking (first antenatal visit documented in the file) and delivery, BMI at booking and delivery, gestational weight gain (GWG) (calculated as weight at delivery minus weight at booking), nationality, parity (number of previous births beyond 24 weeks gestation -divided into nulliparous, multiparous 1-4, and grand multiparous ≥ 4-), history of prior cesarean (yes/no), conception using assisted reproduction, pre-existing medical comorbidities including cardiac conditions, asthma, thyroid disorders, thromboembolism, mental health disorders, chronic hypertension, and diabetes.

The sample was divided into exposed (prior prepregnancy bariatric surgery) and unexposed (no prior bariatric surgery). Additionally, in women with early booking (within the first 20 weeks of gestational age), the GWG was compared to the Institute of Medicine (IOM) guidelines on expected weight gain during pregnancy for different BMI groups at booking^19^. These women were divided into low, adequate, and excess weight gain groups.

The primary maternal outcomes included the incidence of gestational diabetes (GDM), diagnosed with an abnormal 75-gram glucose tolerance test during pregnancy; gestational hypertension (GHT), defined as any new onset of high blood pressure during pregnancy and included pre-eclampsia and associated complications; documented diagnosis of anemia in pregnancy and cholestasis of pregnancy; mode of delivery-spontaneous vaginal delivery (SVD), instrumental vaginal delivery (IVD), and cesarean delivery (CD); postpartum hemorrhage (PPH) defined as estimated blood loss ≥ 500ml during VD and ≥ 1000ml during CD; preterm birth (PTB) defined as delivery at less than 37 weeks gestation, including moderate PTB determined as delivery at less than 34 weeks gestation. The GWG was also analyzed as an outcome (calculated as the weight at delivery minus weight at booking).

The neonatal outcomes included birthweight (BW) in grams; customized BW centiles; low birth weight (LBW) defined as birthweight less than 2500g and were considered in PTB and term births separately; macrosomia defined as birthweight more than 4000g; small for date (SFD) and large for date (LFD) specified as customized BW centiles ≤ 10% and ≥ 90% respectively; incidence of meconium-stained liquor, congenital anomalies; neonatal intensive care (NICU) admission and low APGAR score (Appearance, Pulse, Grimace, Activity, and Respiration) score at 5 minutes of life.

These outcomes were also compared in the three GWG groups separately for the exposed and unexposed groups to explore the relationship between GWG during pregnancy and perinatal outcomes and evaluated if this association was different in women who had prior bariatric surgery compared to those who did not.

### Statistical analysis

Continuous variables were reported as mean ±  standard deviation (SD) or median ±  interquartile range (IQR) based on the variable distribution, which was assessed using histograms and the Shapiro-Wilk test. Categorical variables were presented as frequencies and percentages. Demographic comparisons utilized an unpaired t-test for continuous variables and Chi-Square or Fisher's exact for categorical variables as appropriate.

Binary outcomes in the two exposure groups were analyzed using logistic regression models to obtain crude odds ratios (ORs) and 95% confidence intervals (CIs). For gestational diabetes analysis, women with pre-existing diabetes were excluded. Adjusted estimates (aOR) were derived by incorporating relevant demographic variables in the model as potential confounders, including age, parity, BMI at booking, weight gain, pre-existing disorders, assisted reproduction, and nationality. Additionally, the model for the delivery mode was adjusted for a previous CD, with Wald p-values reported. Birthweight and GWG were analyzed using linear regression, and both crude and adjusted coefficients were reported. Birthweight centiles were assessed using the Wilcoxon rank-sum test due to non-Gaussian distribution.

Binary outcomes in the GWG groups were compared using Fisher's exact test or Chi-square as appropriate. Birthweight and birthweight centiles were compared using one-way ANOVA and the Kruskal-Wallis test. The extracted data were checked for missing values, and a complete case analysis was performed if less than 10% were missing in each exposure group. Evidence against the null hypothesis of no difference was considered if the p-value was less than 0.05. All statistical analyses were done in STATA, edition 18.0.

## Results

During the study period, there were 194 women with a history of bariatric surgery before pregnancy (exposed) and 8129 women who did not (unexposed). Table 1 shows the demographic variables in the two groups. Maternal age and parity were similar between the groups, with the mean age being 30 years and more than 65% multiparous.

Women in the exposed group were statistically significantly shorter and had a lower booking weight compared to the unexposed group (Table 1). The mean BMI at booking was similar between the groups; however, unexposed women were more likely to have a BMI defined as obese compared to those exposed at booking. Women with previous bariatric surgery did not have a statistically significant difference in BMI at delivery. Still, they demonstrated statistically more significant mean (SD) weight gain during pregnancy, 8.37 kg ( ± 5.6) versus 6.55 kg ( ± 6.3), p <  0.001. The difference between the groups was statistically significant for several variables, with the exposed having higher history of previous cesarean delivery, a higher risk of a pre-existing medical illness, and conception by assisted reproduction.

**Figure 1.**
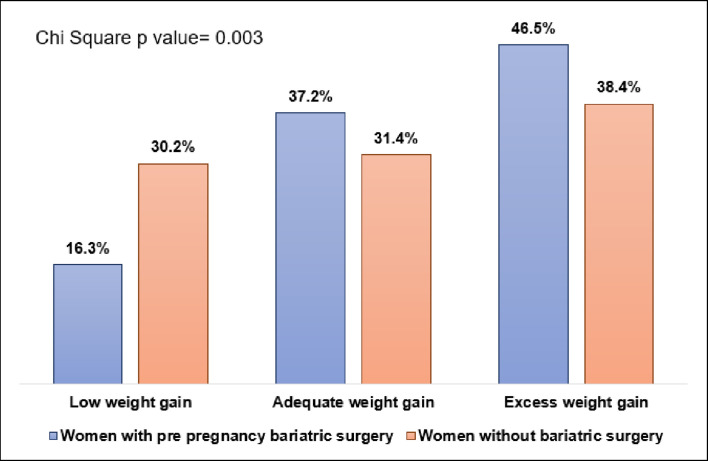
Percentage of women with and without bariatric surgery in each weight gain group.

Of the 4632 women with an early booking visit, 129 (66.5% of the total 194) were exposed, and 4503 (55.4% of the total 8129) were unexposed. Table 2 shows the BMI at booking and delivery and the GWG in the exposure groups divided into the three GWG categories. Nearly 47% of the exposed group had excess GWG during pregnancy compared to 38% in the unexposed group, as shown in Figure 1. Only 16% of the exposed had low GWG compared to 30% in the unexposed (p-value for comparison between exposed and unexposed in all three GWG groups = 0.003).

Maternal outcomes are shown in Table 3. After adjusting for confounders, including BMI at booking, the mean GWG in the exposed group was 1.33 kg more than the mean GWG in the unexposed group (95% CI 0.55-2.13, p = 0.001). Nearly 33% of women developed gestational diabetes equally in both groups. No statistically significant difference was noted in the incidence of anemia and cholestasis of pregnancy. Almost 5% of the exposed developed gestational hypertension compared to 3% of the unexposed; the difference displayed as not statistically significant. After adjusting for confounders, the exposed group had 1.78 times the odds of having PTB (aOR 1.78 95%CI 1.16-2.74, p = 0.008) and 2.2 times higher odds of delivering at less than 34 weeks gestation (aOR 2.20 95%CI 1.00-4.84 p = 0.049).

There was a statistically significant difference in the mode of delivery between the two groups, with nearly 80% of the exposed group delivering by CD compared to only 36% in the unexposed (aOR 6.52 95%CI 4.28-9.93; p < 0.001). Among those who delivered vaginally, the exposed group also had three times higher odds of instrumental delivery (aOR 3.11 95%CI 1.43-6.77, p = 0.004). The increased number of CDs was reflected in the statistically significant increase in the median estimated blood loss at delivery in the bariatric patients (p < 0.001); however, there was no difference in the incidence of PPH.

Neonatal outcomes in the two groups are outlined in Table 4. The BW was significantly lower in the exposed group; after adjusting for confounders, the mean BW in the exposed group was 80.4g less than the unexposed (95% CI -153.0, -5.8; p = 0.034). However, the groups had no statistically significant difference in the median customized BW centiles. For babies who were delivered at term ( ≥ 37 weeks), the exposed group had 2.6 times higher odds of being LBW (aOR 2.60 95%CI 1.34-5.03, p = 0.005). No statistically significant difference was noted in the incidence of macrosomia, LFD babies, or meconium-stained liquor.

There were significantly more congenital abnormalities in the exposed group, 3.6% versus 1.4% in the unexposed (p = 0.017). After adjusting for confounders, there were 2.64 times higher odds of congenital anomalies in women with previous bariatric surgery (aOR 2.64 95%CI 1.21-5.77, p = 0.015). 16.5% of babies in the exposed group were admitted to the NICU compared to 11.8% in the unexposed; however, this association was insignificant after adjusting for confounders. The babies born to bariatric women were significantly more likely to have an Apgar of less than seven at five minutes (aOR 3.75 95%CI 1.12-12.5, p = 0.032).

The risks of various perinatal outcomes were compared among the three GWG groups, as shown in Table 5. In the bariatric group, there was no statistically significant difference in GDM, GHT, PTB, CD, congenital anomalies, or NICU admission concerning the adequacy of GWG. Significantly more women in the low GWG category had LBW babies (28.6%, p = 0.033); the mean BW and median BW centiles were lowest in this same category (2775 grams versus 3289 grams in the high GWG group, p = 0.004 and 57.5% versus 74.5% in the high GWG group, p = 0.040, respectively). The unexposed group showed contrast, where the inadequacy of GWG additionally affected outcomes such as GDM, GHT, and preterm birth.

## Discussion

This study demonstrates that women with previous bariatric surgery, when compared to women without prepregnancy bariatric surgery, gained significantly more weight during their pregnancies. There was a significant increase in the risk of cesarean delivery and preterm birth in the bariatric group. This group also had babies with significantly lower birth weight, a higher probability of low birth weight babies at term, congenital anomalies, NICU admission, and low Apgar scores at five minutes. In this group, women who gained lower than expected weight during their pregnancy had a higher risk of low birth weight babies and had lower birthweight and birthweight centiles. All other outcomes were similar regardless of the adequacy of the weight gain. The results provide compelling insights into pregnancy outcomes following bariatric surgery and the influence of gestational weight gain.

### Comparison with existing literature

Pregnant women in this study with a history of bariatric surgery showed significantly higher GWG. In women with early booking, nearly 47% had excess GWG, while 37% gained adequate weight. This group performed better than the control group regarding less than appropriate GWG; however, previous studies report no difference in the GWG in pregnant women post-bariatric surgery.

A systematic review concluded that when BMI-matched pregnant cohorts were compared, bariatric surgery before pregnancy did not affect total maternal gestational weight gain^20^. A Canadian study in 2022 reported that there is less than adequate trimester-specific and overall GWG in pregnancies post-bariatric surgery^21^; however, this was a much smaller study with only 63 post-surgery pregnant women who underwent a malabsorptive procedure (contrasting with the restrictive one in this study's cohort). A 2023 published prospective UK study followed the GWG in 100 women with prior bariatric surgery who were early pregnancy BMI-matched to 100 women who did not have a previous surgery, reporting no statistically significant difference in the GWG of the post-bariatric surgery group^22^; however, this study did not adjust for other maternal variables and had a smaller sample size. It is to be noted that many variables, such as the type of bariatric surgery, length of time between surgery and conception, and the genetic susceptibilities of the population under study, may impact this and, as such, may explain the results of this study which was in a setting where restrictive rather than malabsorptive bariatric surgeries are common. Despite significant healthcare measures, obesity is still rising at an alarming rate.

Maternal and neonatal outcomes associated with prepregnancy bariatric surgery have been well-reported in the past. Evidence from extensive studies, including 670 women post bariatric surgery, shows that they have a lower risk of GDM and LFD babies^20^. This study was performed in a population reporting a GDM risk of only 6.8%; this is very different from the Qatar population, which has a chance of GDM as high as 21.5%^23^. Additionally, this study considered women in the obesity category in their pregnancies, which increases the GDM risk of the entire cohort by 17% points, as Bashir et al. reported^23^, which accounts for the high risk of GDM in this paper, equally affecting both groups. Despite bariatric surgery, the risk of GDM still exists for these women.

Gestational hypertension was higher in the post-bariatric surgery group of this study, although this finding was not statistically significant. Previous studies that include a systematic review indicate a reduced risk of gestational hypertension in women following surgery^14,24,25^, with varying outcomes observed in BMI-matched comparison groups^20^. Bariatric surgery effectively reduces obesity, and it could follow that this would confer benefits in terms of lower risk of hypertensive disorders. However, this study included only women with obesity despite bariatric surgery, which could explain the higher risk of GHT reported. Similarly, previous reviews show a higher risk of anemia in the exposed group^26^, which is not seen in this study- the difference in the type of bariatric procedure could justify this.

This paper reports a very high risk of CD and instrumental delivery in the category of exposed women. There are conflicting reports in the literature around cesarean section rates in post-bariatric surgery pregnancies. A past meta-analysis showed inconsistent literature regarding CD incidence after bariatric surgery^14^. Other systematic reviews and meta-analyses showed a 40-50% decreased odds of CD in post-bariatric surgery pregnancies^24,25^. There is, therefore, a need for robust studies to explore these rates. This study found that higher odds of CD exist even after adjusting for the history of previous cesarean. Women who have undergone bariatric surgery may have been more inclined to choose elective CD, which could explain the much higher risk in this group. The mean maternal blood loss at delivery was significantly higher in the bariatric surgery group in this study, likely due to the increase in CD in this group. The increased odds of PTB in the exposed group of this study are similar to what has been reported in previous systematic reviews^14,15^.

The findings of the neonatal outcomes in this research are much more consistent with past literature. The higher odds of congenital anomalies align with the results of a recent meta-analysis, which identified ten studies reporting the same^15^. However, other studies found no significant difference for congenital anomalies^27^. The increased risk of congenital anomalies is possibly associated with the nutrient and vitamin deficiencies that arise in women who have had a bariatric procedure compounded when they become pregnant.

Most reviews report a lower birthweight in the exposed group and a higher risk of SFD baby and NICU admission. A recent systematic review comparing obese pregnant women with a history of bariatric surgery to BMI-matched pregnant women without a history of bariatric surgery showed a significant increase in the incidence of SFD, as well as significantly lower birth weights in pregnancies following bariatric surgery^14^, supported by further systematic reviews showing the reduced infant birth weight in this group^20^. Studies show that GWG is not associated with this increased risk of SFD babies, as demonstrated in this paper^22^. There is a higher risk of LBW babies, specifically in term babies, in this research. This analysis was done to eliminate the impact of increased PTB in this group. The implications for fetal weight could again be due to the nutritional deficiencies associated with bariatric procedures. Additionally, some studies report that fluctuations in blood glucose levels occur in pregnancy after bariatric surgery, which can impact fetal growth^28^.

### Strengths and limitations

This study uses registry data collected meticulously by independent and trained data extractors, providing good-quality data with minimal missing data. This paper is the first study from Qatar exploring the impact of GWG on pregnancy outcomes in women with prepregnancy bariatric surgery. We report some exciting findings that contrast with what was documented previously. The population in Qatar is very diverse and multiethnic, with women from more than 90 countries delivering at the tertiary hospital. It is, therefore, challenging to extrapolate results from studies from around the world to the population in Qatar. The results from this study will help counsel the women in Qatar regarding the complications associated with pregnancy after bariatric surgery, the preventive measures that can be taken, and the provision of appropriate risk-specific maternity care.

Some limitations need to be taken into consideration. The results only apply to women falling in the obesity category during their pregnancy delivering singleton live births. Pregnancies ending with intrauterine fetal demise were excluded, and this might have underestimated some of the risks. The time interval since gastric surgery was not recorded, and the weight change from surgery to conception was unavailable. There is a wide disparity in group numbers; some negative results could be attributed to a lack of power. Although the most common confounders have been accounted for, there is a chance for residual confounding. For example, nationality alone does not account for the genetic variation existing due to ethnicity (data about ethnicity was unavailable separately). A smaller number of women had an early booking in the hospital- some of the non-significant results noted in the association between GWG and the outcomes could be because of the smaller numbers. However, the results concur with past publications.

## Conclusions

With the exponential rise in obesity rates in childbearing women and the use of bariatric surgery as a management strategy, the implications for subsequent pregnancy have to be considered and are gaining relevance and importance. The findings of this study highlight differences in perinatal outcomes such as preterm birth, low birth weight, congenital anomalies, cesarean deliveries, and gestational weight gain between post-bariatric surgery women and controls. These insights can help inform the planning and provision of appropriate maternity care to enhance patient safety and outcomes.

### Acknowledgements

We take this opportunity to acknowledge the contributions of the PEARL-Peristat Study team- Dr. Stephen Lindow and Mr. Frank Tuennisen - for providing a high-quality dataset for this study and their valuable statistical input.

### Conflict of interest

The authors have no conflicts of interest to declare, which could have influenced the information in this paper.

### Funding

There was no funding required to conduct this study.

## Figures and Tables

**Table 1:** Maternal demographics in the comparison groups.

Demographics		No prior Bariatric surgery N = ,129		Prepregnancy Bariatric surgery N = 194		P-value
		n	% of N	n	% of N	
Maternal age in years (Mean ± SD)		30.4 ± 5.5		30.6 ± 5.4		0.886
Maternal age	< 30 years	3491	42.9	78	40.2	0.446
	> = 30 years	4638	57.1	116	59.8	
Maternal height in cm (Mean ± SD)		158.7 ± 5.9		157.6 ± 5.9		0.008
Maternal weight at booking kg (Mean ± SD)		82.2 ± 13.7		79.9 ± 14.9		0.023
Maternal BMI at booking kg/m^2^ (Mean ± SD)		32.6 ± 4.9		32.1 ± 5.2		0.376
BMI at booking categories	< 25 kg/m^2^	194	2.4	2	1.0	
	25-24.99 kg/m^2^	2305	28.4	78	40.2	0.001
	≥ 30 kg/m^2^	5630	69.3	114	58.8	
Maternal BMI at delivery kg/m^2^ (Mean ± SD)		35.2 ± 4.3		35.5 ± 4.5		0.176
Weight gain in pregnancy (kg) (Mean ± SD)		6.55 ± 6.3		8.37 ± 5.55		< 0.001
Maternal nationality- Qatari		2876	35.4	69	35.6	0.957
Maternal parity	Nulliparous	1794	22.1	53	27.3	
	Multiparous	5499	67.6	127	65.5	0.120
	Grand multiparous	836	10.3	14	7.2	
History of previous caesareans		2287	28.1	100	51.5	< 0.001
Assisted Reproduction		252	3.1	13	6.7	0.005
Preexisting medical history		1423	17.5	49	25.3	0.005
Chronic hypertension		39	0.5	6	3.1	< 0.001
Preexisting diabetes		267	3.3	12	6.2	0.027

SD- standard deviation; BMI- body mass index; Pre-existing medical history includes cardiac conditions, asthma, thyroid disorders, thromboembolism, mental health disorders, chronic hypertension, and pre-existing diabetes; Continuous variables analyzed using t-test; categorical variables analyzed using Chi-square; p < 0.05 is considered evidence against the null hypothesis

**Table 2:** Classification as per weight gain during pregnancy in women with early booking

Women having a booking visit N = 4632		Adequate weight gain	Low weight gain	Excess weight gain
Women with prepregnancy	BMI at booking	31.4 ± 4.6	34.0 ± 5.5	30.0 ± 4.8
bariatric surgery	BMI at delivery	34.9 ± 4.3	35.1 ± 5.1	35.6 ± 4.3
N = 129	Weight gain (kg)	8.6 ± 1.6	2.8 ± 2.0	14.2 ± 3.6
Women with no prior	BMI at booking	31.5 ± 4.6	34.1 ± 4.7	30.2 ± 4.6
bariatric surgery	BMI at delivery	34.8 ± 4.3	35.0 ± 4.4	36.0 ± 4.3
N = 4503	Weight gain (kg)	8.2 ± 2.0	2.0 ± 2.9	14.8 ± 4.2

BMI- body mass index; BMI and weight gain are reported in mean and standard deviation; Category of weight gain determined according to Institute of Medicine guidelines for adequate weight gain in pregnancy according to maternal BMI at booking

**Table 3:** Comparison of maternal outcomes between the groups

Maternal Outcomes	No prior BariatricN = 8,129 (baseline)		Prepregnancy BariatricN = 194		Crude Coefficients/ ORs (95% CI)	P-value	Adjusted Coefficients/ ORs (95%CI)	P-value
	n	% of N	n	% of N				
Gestational weight gain* (Mean ± SD)	6.55 ± 6.3		8.37 ± 5.55		1.81 (0.91-2.71)	< 0.001	1.33 (0.55-2.13)	0.001
Gestational diabetes #	2629	33.4	60	33	0.98 (0.72-1.33)	0.894	1.03 (0.75-1.42)	0.862
Gestational hypertension	265	3.3	10	5.2	1.61 (0.84-3.08)	0.148	1.45 (0.75-2.80)	0.266
Anaemia	963	11.9	25	12.9	1.10 (0.72-1.68)	0.658	1.13 (0.74-1.74)	0.562
Cholestasis of pregnancy	143	1.8	3	1.6	0.88 (0.28-2.78)	0.823	0.84 (0.26-2.67)	0.768
Preterm birth ( < 34 weeks gestation)	132	1.6	7	3.6	2.27 (1.05-4.92)	0.038	2.20 (1.00-4.84)	0.049
Preterm birth ( < 37 weeks gestation)	638	7.9	26	13.4	1.82 (1.19-2.77)	0.005	1.78 (1.16-2.74)	0.008
Cesarean delivery (compared to all vaginal births)	2924	36	155	79.9	7.07 (4.97-10.08)	< 0.001	6.52 (4.28-9.93)	< 0.001
Instrumental delivery (compared to spontaneous vaginal)	413	7.9	11	28.2	4.56 (2.25- 9.22)	< 0.001	3.11 (1.43-6.77)	0.004
Estimated blood loss (ml)$ (Median IQR)	300 (200-400)		350 (300-475)		-	< 0.001	-	-
Postpartum haemorrhage$	379	4.7	9	4.7	1.00 (0.51-1.96)	0.996	0.95 (0.48-1.87)	0.873

*GWG coefficients estimated using linear regression; Instrumental birth analyzed after excluding CDs; SD- standard deviation; CI- confidence interval; OR- odds ratios; p < 0.05 is considered evidence against null hypothesis; Odds ratios calculated using logistic regression models, Wald p-values reported; Adjustment done for age, parity, BMI at booking, weight gain, pre-existing disorders, assisted reproduction and nationality, for the mode of delivery- additionally adjusted for previous cesarean; #- women with pre-existing diabetes (279) excluded; $- missing data =  59 ( < 1%)

**Table 4:** Comparison of fetal outcomes between the groups

Fetal Outcomes	No prior Bariatric N = 8,129 (baseline)		Prepregnancy Bariatric N = 194		Crude Coefficient/ORs (95% CI)	P-value	Adjusted Coefficient/ORs (95% CI)	P-value
	n	% of N	n	% of N				
Birthweight (grams)* (Mean ± SD; coefficients)	3255 ± 521		3174 ± 593		-80.4 (-154.8, -5.9)	0.035	-79.4 (-153.0, -5.8)	0.034
Birthweight centiles (%) # (Median IQR)	65.5 (47.2)		70.7 (47.9)		-	0.371	-	-
Low birth weight in preterm (birthweight < 2500g)	317	3.9	13	6.7	1.01 (0.46-2.22)	0.975	1.02 (0.45-2.28)	0.967
Low birth weight at term (birthweight < 2500g)	184	2.3	10	5.2	2.51 (1.30-4.84)	0.006	2.60 (1.34-5.03)	0.005
Small for date baby# ( < 10th centile)	334	4.5	14	7.7	1.78 (1.02-3.10)	0.042	1.71 (0.95- 3.09)	0.075
Macrosomia (>4000g)	483	5.9	6	3.1	0.51 (0.22-1.14)	0.102	0.53 (0.23-1.20)	0.129
Large for date baby (>90th centile)	1508	20.2	40	21.2	1.11 (0.78-1.59)	0.554	0.98 (0.66-1.45)	0.920
Meconium-stained liquor	751	9.2	16	8.3	0.88 (0.53-1.48)	0.637	0.88 (0.52-1.47)	0.623
Congenital anomalies	117	1.4	7	3.6	2.56 (1.18-5.57)	0.017	2.64 (1.21-5.77)	0.015
NICU admission	955	11.8	32	16.5	1.48 (1.01-2.18)	0.045	1.41 (0.95-2.08)	0.086
Apgar score < 7 at 5 min $	34	0.4	3	1.6	3.75 (1.14-12.33)	0.029	3.75 (1.12-12.5)	0.032

* Birthweight coefficients obtained using linear regression; NICU- neonatal intensive care unit; CI- confidence interval; OR- odds ratios; p < 0.05 is considered evidence against null hypothesis; Odds ratios calculated using logistic regression models; Adjustment done for age, parity, BMI at booking, weight gain, pre-existing medical disorders, assisted reproduction; Wald p-values reported; #- missing data = 673 (8%); $ missing = 11 ( < 1%)

**Table 5:** Risks of the perinatal outcomes according to the category of weight gain during pregnancy

Outcomes	Prepregnancy Bariatric Surgery; N = 129				No prior Bariatric surgery; N = 4503			
	Adequate weight gain 48 (37.2%) n, %N	Low weight gain 21 (16.3%) n, %N	Excess weight gain 60 (46.5%) n, %N	P-value	Adequate weight gain 1412 (31.4%) n, %N	Low weight gain 1361 (30.2%) n, %N	Excess weight gain 1730 (38.4%) n, %N	P-value
Gestational diabetes*	20 (45.5%)	8 (44.4%)	15 (27.3%)	0.134	490 (36.3%)	654 (51.2%)	442 (26.4%)	< 0.001
Gestational hypertension	2 (4.2%)	1 (4.8%)	4 (6.7%)	0.876	52 (3.7%)	24 (1.8%)	75 (4.3%)	< 0.001
Preterm birth ( < 34 weeks)	3 (6.3%)	2 (9.5%)	1 (1.7%)	0.461	27 (1.9%)	48 (3.5%)	18 (1.0%)	< 0.001
Preterm birth ( < 37 weeks)	10 (20.8%)	5 (23.8%)	8 (13.3%)	0.392	136 (9.6%)	159 (11.7%)	118 (6.8%)	< 0.001
Caesarean birth	43 (89.6%)	16 (76.2%)	47 (78.3%)	0.226	542 (38.4%)	547 (40.2%)	625 (36.1%)	0.066
Low birth weight	6 (12.5%)	6 (28.6%)	4 (6.7%)	0.033	90 (6.4%)	134 (9.8%)	84 (4.9%)	< 0.001
Small for date	2 (4.3%)	3 (15.8%)	4 (7.3%)	0.257	65 (4.9%)	52 (4.0%)	65 (4.1%)	0.429
Congenital anomalies	3 (6.3%)	1 (4.8%)	3 (5.0%)	1.000	20 (1.4%)	24 (1.8%)	22 (1.3%)	0.519
NICU admission	11 (22.9%)	6 (28.6%)	10 (16.7%)	0.474	168 (11.9%)	179 (13.2%)	197 (11.4%)	0.317
Birthweight (kg) (Mean SD)	3107 (549)	2775 (805)	3289 (562)	0.004	3233 (527)	3132 (553)	3323 (553)	< 0.001
Birthweight centiles (Median IQR)	73.9 (40.9)	57.5 (62.9)	75.4 (46)	0.040	65.5 (47)	63.7 (47.3)	69.9 (46.7)	< 0.001

* Analysis of GDM excludes women with pre-existing diabetes; NICU: neonatal intensive care; SD- standard deviation; IQR- interquartile range; Fisher's and Kruskal Wallis tests used for analysis; Category of weight gain determined according to IOM guidelines for adequate weight gain in pregnancy according to maternal BMI at booking

**Table:** List of abbreviations

ANOVA	Analysis of variance
aOR	Adjusted odds ratios
BMI	Body mass index
BW	Birthweight
CD	Cesarean delivery
CI	Confidence intervals
GDM	Gestational diabetes mellitus
GHT	Gestational hypertension
GWG	Gestational weight gain
IOM	Institute of Medicine
IQR	Interquartile range
IRB	Institutional review board
IVD	Instrumental vaginal delivery
LBW	Low birthweight
LFD	Large for date
NICU	Neonatal intensive care unit
OR	Odds ratios
PEARL	Perinatal Neonatal Outcomes Research Study in the Arabian Gulf
PPH	Postpartum hemorrhage
PTB	Preterm birth
SD	Standard deviation
SFD	Small for date
SVD	Spontaneous vaginal delivery
